# Urinary Exosomes Contain MicroRNAs Capable of Paracrine Modulation of Tubular Transporters in Kidney

**DOI:** 10.1038/srep40601

**Published:** 2017-01-17

**Authors:** Tannia Gracia, Xiaonan Wang, Ya Su, Elizabeth E. Norgett, Timothy L. Williams, Pablo Moreno, Gos Micklem, Fiona E. Karet Frankl

**Affiliations:** 1 Department of Medical Genetics, Cambridge Institute for Medical Research, University of Cambridge, Cambridge, UK; 2 Department of Pathology, University of Cambridge, Cambridge, UK; 3 Department of Veterinary Medicine University of Cambridge, Cambridge, UK; 4 Departments of Genetic, University of Cambridge, Cambridge, UK

## Abstract

Exosomes derived from all nephron segments are present in human urine, where their functionality is incompletely understood. Most studies have focused on biomarker discovery rather than exosome function. Through sequencing we identified the miRNA repertoire of urinary exosomes from healthy volunteers; 276 mature miRNAs and 345 pre-miRNAs were identified (43%/7% of reads). Among the most abundant were members of the miR-10, miR-30 and let-7 families. Targets for the identified miRNAs were predicted using five different databases; genes encoding membrane transporters and their regulators were enriched, highlighting the possibility that these miRNAs could modulate key renal tubular functions in a paracrine manner. As proof of concept, cultured renal epithelial cells were exposed to urinary exosomes and cellular exosomal uptake was confirmed; thereafter, reduced levels of the potassium channel ROMK and kinases SGK1 and WNK1 were observed in a human collecting duct cell line, while SPAK was unaltered. In proximal tubular cells, mRNA levels of the amino acid transporter gene *SLC38A2* were diminished and reflected in a significant decrement of its encoded protein SNAT2. Protein levels of the kinase SGK1 did not change. Thus we demonstrated a novel potential function for miRNA in urinary exosomes.

Urinary exosomes are lipid membrane-bound nanovesicles released from intracellular multivesicular bodies (MVBs)^1^,^2^ and derived from all cells in the urinary tract^3^,^4^,^5^. During the inward budding of endosomes that give origin to exosomes, proteins^2^, mRNAs^6^, microRNAs (miRNAs)^7^, noncoding RNA (ncRNA)^8^, transcription factors^9^ and other biomolecules present in the cytosol can be incorporated. The lipid bi-layer of these nanovesicles provides the cargo with stable storage conditions and protects it from degradation by extracellular proteases and ribonucleases^10^. Studies in other tissues have shown that once exosomes and other microvesicles are released into the extracellular environment, interactions with cells can occur by direct ligand-receptor signalling, by exosomal fusion to the target cell membrane and discharge of exosomal content directly into the cytoplasm, or via phagocytosis/macropinocytosis^11^,^12^. Exosomes are known to deliver biologic cargo not only to neighbouring cells but also long distance^13^.

The majority of studies concerning urinary exosomes have focused on their potential as biomarkers of disease pathology and progression, including prostate and bladder cancers^14^,^15^,^16^,^17^, but their functional significance is now being addressed. Inter-cellular signalling by exosomes in cultured murine renal epithelial cells was demonstrated for the first time by Street *et al*.^18^, who suggested that collecting duct cell-derived exosomes can transfer the ability to express AQP2. Our previous studies revealed that urinary exosomes inhibit bacterial growth of both commensal and uropathogenic *E. coli* by inducing bacterial lysis^19^. Bruschi and colleagues demonstrated that urinary exosomes can consume oxygen in order to synthesize ATP aerobically, thus suggesting metabolic activity^20^, and Jiang *et al*. showed that urinary exosomes derived from stem cells may have the potential to inhibit podocyte apoptosis and promote vascular regeneration in the kidney^21^.

Various protein compendia of urinary exosomes have been reported^3^,^5^,^19^,^22^,^23^,^24^,^25^,^26^, but the identification of their miRNA content has been limited to one sequence based study^10^, and three studies utilising RT-qPCR or microarray approaches that restrict the study to a specific set of known miRNAs^7^,^15^,^27^. The lack of efficient methods to isolate small RNAs from vesicles has been a constraint to miRNA profiling, and there is no consensus regarding the optimal method for isolation of exosomes. Here we aimed to identify the miRNA repertoire of urinary exosomes from healthy individuals using the most efficient methods available for exosomal isolation and small RNA extraction. We also investigated the direct effects of exosomal miRNA on gene expression and protein levels of selected predicted targets in kidney-derived cell lines.

## Results

### Urinary exosome isolation and miRNA extraction

Exosomes from the second morning urine void of healthy volunteers on no regular medication and with no urinary dipstick abnormalities (aged 32–46 y) were isolated as previously described^19^. Electron microscopy images showed the expected intact vesicles of diameter 59.0 ± 1.93 nm; 92% of the population fell in the 30–80 nm range (Supplementary Figure S1). Western blots confirmed the presence of the exosomal markers CD63 and TSG101 in these isolated vesicles but not in exosome-depleted urine (Supplementary Figure S1). The uromodulin content of these preparations was 10–30% of the total protein.

**Figure 1:**
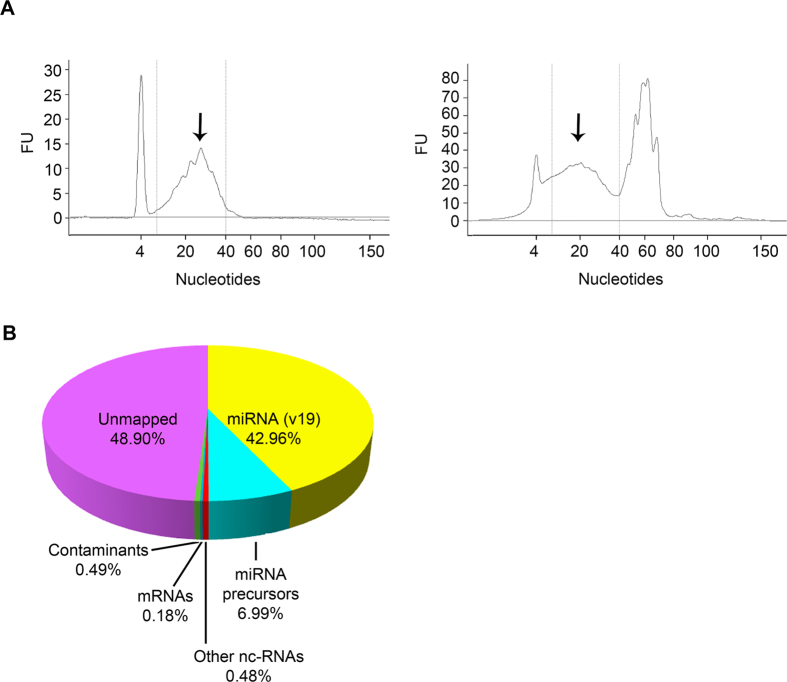
Characterization of the small RNA content of urinary exosomes. (**A**) Bioanalyzer profiles of small RNAs isolated from urinary exosomes of two different volunteers analysed on Small RNA chip. Both profiles exhibited enrichment for the miRNA region (arrowed). (**B**) Deep sequencing analysis of the small RNA fraction isolated from pooled urinary exosomes. Mature microRNAs and pre-microRNAs together comprised 50% of total reads.

We successfully purified and quantified exosomal small RNAs. Electropherograms showed characteristic peaks of both miRNA and other non-coding RNA (ncRNA) species (Fig. 1A). The profiles varied somewhat among subjects, particularly over the size range 40–100 nucleotides (which includes pre-miRNAs, tRNAs, rRNAs and snoRNAs). Amounts of miRNA packaged in the exosomes were 7.3 ± 1.7 ng per 100 ml of urine, and the proportion of miRNA compared to other small RNAs was 50.4 ± 5.7% (Supplementary Table S1). There were no significant differences between male and female subgroups (p = 0.29).

### Next Generation Sequencing

After adapter trimming and quality filtering, just over 10^7^ reads were obtained and aligned to miRBase V19. 0.49% corresponded to sequences in the *E. coli* genome (*E. coli K-12 MG1655)* and were discarded. 43% corresponded to known miRNA sequences, identifying 1197 miRNAs (Fig. 1B). A further 7% corresponded to pre-miRNAs. Those with fewer than 5 reads/million were discarded; 276 mature miRNAs and 345 pre-miRNAs were retained for further analysis (Supplementary Tables S2 and S3). Table 1 shows the 10 most abundant miRNAs and pre-miRNAs. Five of these were also reported in the top 10 most abundant miRNAs and pre-miRNAs in the only other sequence-based analysis of this type of exosome^10^.

After miRNAs and their precursors, other RNA types accounted for a very small fraction of all mappable sequences; 0.28% were tRNAs, while snoRNA/snRNA, lincRNA and rRNA were only represented by 0.01% each. Misc_RNA (defined as any ncRNA that cannot be categorised as anything else) covered 0.17% of the total. Fragments of coding RNA sequences represented a further 0.18%. Almost half of the total reads were classified as unmapped (no match to sequences of *E. coli*, human coding RNA, miRNAs, pre-miRNAs, or other ncRNAs), likely due to the strict sequence alignment parameters employed.

### RT-qPCR validation of urinary exosome miRNAs

**Figure 2:**
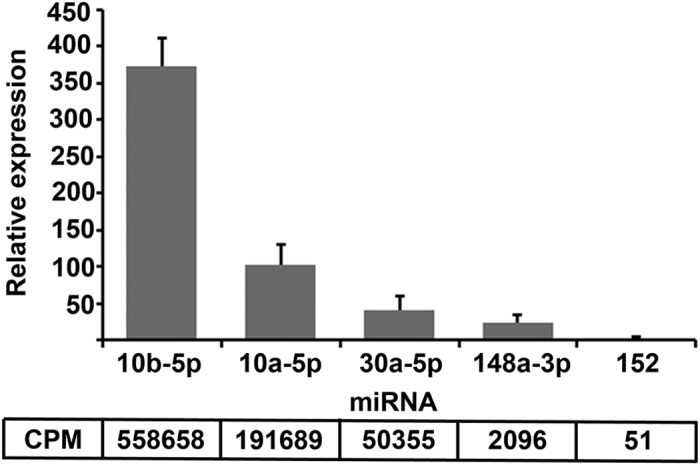
Validation of urinary exosomal miRNAs by RT-qPCR. The expression of selected miRNAs identified by deep sequencing with abundances ranging from 50 to 5.6 × 10^5^ CPM was analysed using sno-RNU6B for normalization. Comparative expression values are in agreement with the relative abundance ratios suggested by the sequencing data. Bars represent normalized expression mean ± SE of at least three independent replicates.

Five miRNAs with different read counts ranging from 50 to 5.6 × 10^5^ counts per million (CPM) were selected for RT-qPCR validation assays: miR10a, miR10b and miR30a (abundant), and miR-148a-3p and miR-152 (low abundance). Melting curve analyses showed specific amplification with no primer dimer or non-specific products. As shown in Fig. 2, expression levels of miR-10b-5p and miR10a-5p were 9 and 2.5 fold higher than miR30a-5p respectively, and all five were in agreement with the relative abundance ratios suggested by the sequencing data.

### miRNA target prediction

We next investigated potential roles of the ten most abundant exosomal miRNAs by inferring their target relationships. Interrogation of five databases for target prediction (MiRBase^28^, miRanda^29^, PicTar^30^, PITA^31^ and miRDB^32^), with acceptance of results present in three or more databases, yielded a target list of genes that were ranked by average of the co-expression scores generated from each of the three databases.

1588 potential targets were identified; 66 of these are known kidney transporters in both proximal and distal nephron. Examples include several members of the solute carrier family (*SLC*), which transport amino acids, glucose and charged and uncharged molecules. Among the most frequently targeted were *SLC38A2*, which encodes the amino acid transporter SNAT2, and the Ca^2+^ transporting ATPase gene *ATP2B1*, targeted by six and four of the ten most abundant miRNAs, respectively. Genes involved in regulation of essential renal physiological processes such as kinases WNK1, SPAK and SGK1 were also frequently represented potential targets. WNK1 and SPAK were targets of at least 3 of the most abundant miRNAs (Supplementary Table 4).

Significant gene enrichment analysis of the target list using DAVID^33^ suggested potential regulatory roles of miRNAs found in urinary exosomes. Gene ontology terms highlighted the possibility for these miRNAs to exert effects on protein kinases and other regulators of key functions such as ion transport in the nephron (Table 2). This was supported by Kyoto Encyclopedia of Genes and Genomes (KEGG)^34^ pathway enrichment analysis, where predicted targets for the most abundant miRNAs were significantly associated with tubular transport, endocytosis, ubiquitin mediated proteolysis and focal adhesion (Table 3). Interestingly, given our previous demonstration of the antibacterial effects of exosomes^19^, the pathway involving bacterial invasion of epithelial cells was among those listed.

### Urinary exosome uptake by cultured human renal epithelial cells

**Figure 3:**
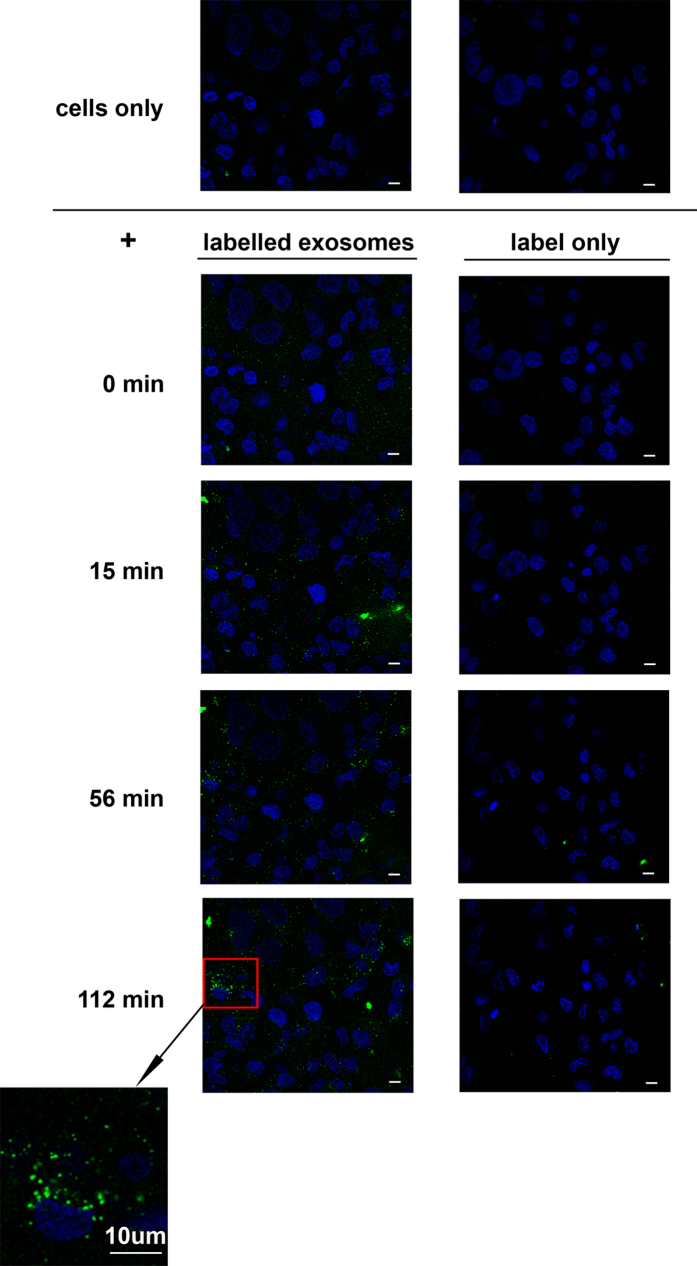
Internalization of urinary exosomes by human collecting duct cells (HCD). Fresh PKH67-labelled urinary exosomes, or PKH67 dye only, were added to HCD cells and observed using cell live microscopy for approximately 2 h. Panels show representative images before (0 minutes) and at various times after exosome or dye addition. Accumulation of labelled exosomes inside cells is clearly observed after 56 minutes. Dye alone did not accumulate in cells. Bars indicate 10 μm.

To test the hypothesis that urinary exosomal miRNA can affect protein expression, we first confirmed cellular uptake of these nanovesicles by cultured renal epithelial cells using live-cell microscopy. Fresh pooled urinary exosomes were surface-labelled with PKH67 and added to human collecting duct cells at 70–80% confluence. Exosomes were demonstrated to become adherent and internalized progressively over a 2 h period of observation; dye alone did not behave in this way (Fig. 3; Supplementary Videos S1 and S2). Cells looked normal after 2 h of live microscopy and were returned to standard culture conditions for another 48 h. At this point the cells had continued to divide to 100% plate confluence, suggesting that exposure to labelled exosomes did not interfere with normal growth.

### Target validation

For proof-of-concept that exosomal miRNA could possibly exert functional effects on cells, we chose to investigate membrane transporters that were in the top 10 predicted target genes, based on KEGG and DAVID analysis. We assessed the effect of exosomal miRNA on the expression of these selected genes in available kidney cell systems by PCR and western blot (Supplementary Figure 2). The selected genes were: *KCNJ1* (encoding potassium channel ROMK1) and *ATP2B1* (encoding plasma membrane calcium-transporting ATPase PMCA1) both expressed in the distal nephron and collecting duct cell line HCD; and *SLC38A2* (encoding amino acid transporter SNAT2), expressed in the proximal tubule and in proximal cell line HKC-8. We confirmed that there were no significant changes using either buffer alone or the prevalent urinary protein, uromodulin, in exposures for both cell lines (Supplementary Figure 3).

**Figure 4:**
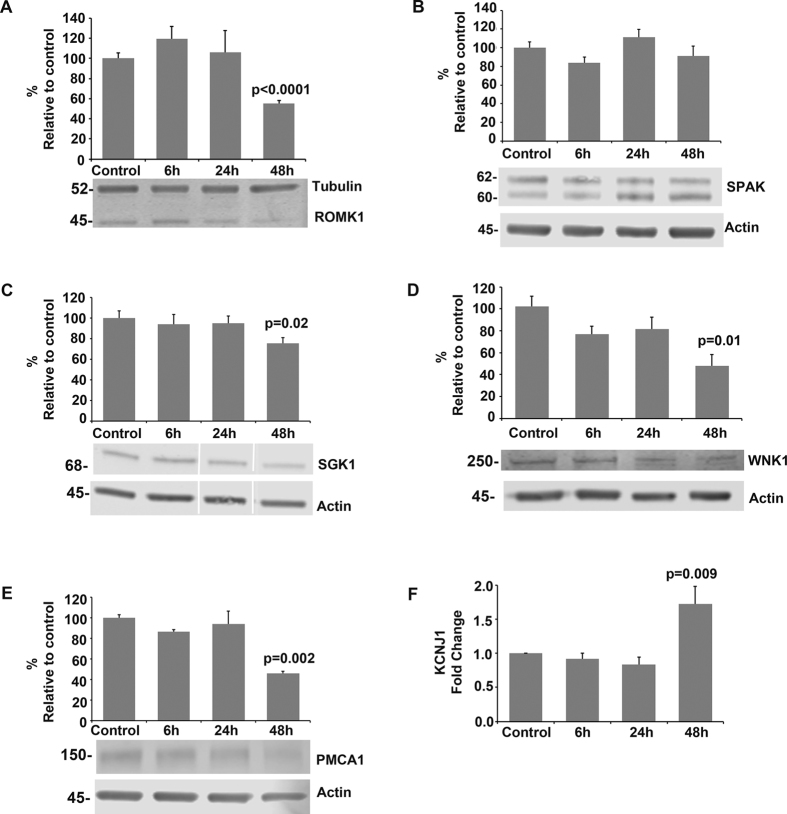
Effects of urinary exosomes on exemplar membrane transporters and regulators in human collecting duct cells (HCD). Panels A–E show western blots and densitometry analyses of HCD cell lysates following exposure of cells to 10 μg of urinary exosomes for 6, 24 or 48 h. (**A**) ROMK1 levels significantly decreased after 48 h exposure; (**B**) SPAK did not change; (**C**) SGK1 and (**D**) WNK1 levels decreased at 48 h. (**E**) PMCA1 was also downregulated. Western blots are representative of three independent repeats for each analysed protein. Graphs show mean ± SE of densitometric intensity. (**F**) *KCNJ1* expression levels in HCD were evaluated by RT-qPCR at the same timepoints. Expression levels decreased by 20% at 24 h but had increased at 48 h of exosomal exposure. Graph represents fold changes compared to control (no exosomes) of three independent repeats.

HCD cells incubated with 10 μg of fresh urinary exosomes for 6, 24 or 48 h showed no changes in cell morphology or confluence. Western blots of cell lysates revealed that protein expression levels of ROMK1 were decreased by nearly half after 48 h of exposure to exosomes (*p* < 0.0001 vs no exosomes, Fig. 4A). To examine the possible contributory effect of relevant kinases we quantified levels of SPAK^35^, SGK1^36^ and WNK1^37^ (Fig. 4B–D). SPAK levels did not change (Fig. 4B), excluding a non-specific effect of exosomes on these cells, while SGK1 and WNK1 were reduced (Fig. 4C and D). Since these have opposing effects on ROMK1, the observed fall in ROMK1 levels is consistent with a direct effect of exosomal miRNA on *KCNJ1.* In support of this hypothesis, gene expression of *KCNJ1*, assessed by qRT-PCR normalized to *GAPDH*, was approximately 20% reduced at 24 h, with recovery by 48 h (Fig. 4F).

In the HCD cells, PMCA1 protein levels also decreased following exosomal exposure by approximately 50% (*p* = 0.002, Fig. 4E). This finding is consistent with a recent report that identified the miR-27 family, present in abundance in our exosomal samples, as down-regulators of *ATP2B1*^38^.

**Figure 5:**
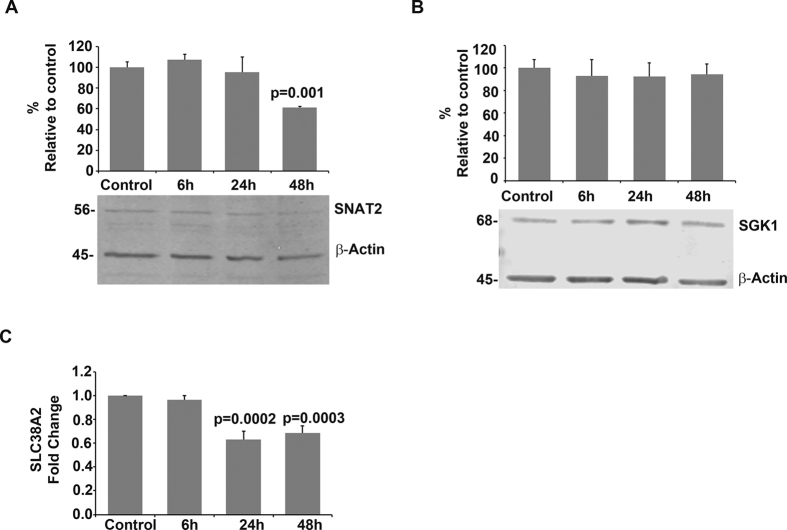
Effects of urinary exosomes on SNAT2 and SGK1 in human proximal tubular cells (HKC-8). Panels A and B show western blots and densitometry analyses of HKC-8 cell lysates following exposure of cells to exosomes for 6, 24 or 48 h. Western blots are representative of three independent repeats for each analysed protein. (**A**) SNAT2 protein levels decreased after 48 h of exposure, whereas (**B**) SGK1 levels did not change. Graphs represent mean ± SE of densitometric intensity. (**C**) The expression of *SLC38A2* in human proximal tubular cells (HKC-8) was evaluated by RT-qPCR at the same timepoints. Downregulation of *SLC38A2* was observed at 24 h of exposure and maintained at 48 h. Graph represents fold changes compared to control (no exosomes) of three independent repeats.

In human proximal tubular cells (HKC-8), SNAT2 levels decreased significantly (*p* = 0.001), without any change in SGK1 (Fig. 5A and B). Expression levels of *SLC38A2* analysed using qRT-PCR and the *B2M* gene (as the appropriate normalizer based on primer efficiency) revealed that gene expression was downregulated by approximately 1/3 after 24 h, and this decrease was maintained after 48 h of incubation with urinary exosomes (Fig. 5C).

## Discussion

Urinary exosomes are being studied intensively to identify biomarkers for renal disease^14^,^39^,^40^,^41^,^42^, alongside an increasing focus on their biological effects^18^,^19^,^20^,^21^. Their functional significance is still incompletely understood and their full potential remains undeveloped. Our initial intention, to produce a catalogue of exosomal miRNAs, gave rise to the opportunity to provide proof of principle that these nanovesicles could be functional. Here, we provide the resulting catalogue, along with preliminary evidence that the miRNAs in urinary exosomes are biologically active and can directly engage in the modulation of protein levels of targeted genes.

The miRNA content of urinary exosomes has been variously reported^7^,^10^,^15^,^27^. We used the most efficient methods for urinary exosome isolation (ultracentrifugation), miRNA extraction (Qiagen miRNeasy-RNeasy)^10^,^43^ and small RNA library construction (Illumina TruSeq small RNA) that were available at the time. Unfortunately, there is no consensus of standard protocols to isolate urinary exosomes and although often only small technical modifications are made, the impact on the final results can be considerable. For example, it is controversial whether or not urine filtration prior to the preparation of urinary exosomes is necessary^44^. Alvarez and co-workers were the first to compare yields of protein, miRNA and mRNA using different methods of urinary exosome isolation^43^. Then, Cheng *et al*. reported exosomal and non-exosomal miRNA populations in urine comparing different miRNA purification methods and using Ion Torrent PGM for sequencing^10^. Although we recovered less miRNA per urine volume than reported by Cheng, the miRNA species identified were similar, with both datasets including miR-10b-5p, miR10a-5p, miR30a-5p, miR26a-5p and miR-30d-5p among the top 10 most abundant miRNAs. Differences in amounts and abundance may be partly explained by Cheng’s use of frozen urine samples and omission of a filtration step; the first might reduce overall exosomal yield because of vesicular lysis, while the second increases the possibility of miRNA coming from cellular material. We demonstrated in preliminary experiments that the apparent quantity of miRNA obtained from urine which was not filtered was much higher when compared with filtered urine from the same source (Supplementary Table S5), suggesting the erroneous inclusion of miRNA from non-exosomal sources in the unfiltered samples. Our exosomes were isolated from fresh samples filtered with a 0.22 μm membrane before the ultracentrifugation step, thus the miRNA we purified will have originated from exosomes and not other cellular sources. Thus we advise filtration to ensure removal of cellular material before ultracentrifugation, which would likely introduce artefacts.

Various algorithms are available to predict miRNA targets^28^,^29^,^30^,^31^,^32^; they vary in their methodologies and levels of stringency for seed pairing and scoring, producing highly distinct results. The relevance of identifying miRNA families is that they point to common sequences or configuration in groups of genes, that suggest a common function^45^. Here we have included five different approaches, and to garner the highest sensitivity/specificity in target selection, we listed targets only if they were predicted by at least 3 of these, which increases the likelihood of predicting true interactions. An important observation from our study is that targets predicted for the members of the miR-10, miR-30 and the let-7 miRNA families, listed among the most abundant, showed enrichment not only for genes encoding transporters of ions and organic molecules in the kidney but also of known regulators, suggesting potential correlations of miRNA families targeting or regulating specific renal functions.

For proof of concept that exosomal miRNAs might have functional effects on their predicted targets, we chose targets represented in the dataset that are expressed in different nephron segments. *SLC38A2*′s encoded protein, the proximal tubule sodium-coupled neutral amino acid transporter SNAT2, mediates cellular uptake of glutamine and other small neutral amino acids^46^ and also mediates increased proline influx under conditions of amino acid deprivation in renal proximal-tubule-like epithelial cells^47^. The reduction in SNAT2 and *SLC38A2* following exosomal exposure was greater than those, typically described as small^48^, exerted by any specific miRNA on any individual target, and this may reflect the whole-urine source of the material used. In animal cells, mature microRNAs can work either by reducing the stability of mRNA or by inhibiting translation, by binding specific sites in their 3′UTRs or truncating translation initiation respectively^49^,^50^. Thus the observed results for this aminoacid transporter could be because of the multiple miRNA binding sites predicted in the same 3′UTR, implying a multiplicative repressive effect^51^,^52^, furthermore, multiple miRNAs of different families are known to act synergistically^53^. Fewer listed miRNAs were identified as potential downregulators of *KCNJ1,* which functions in the distal nephron, although the protein repression we observed was similar. Unlike *SLC38A2,* decreased *KCNJ1* gene expression levels following exosomal exposure recovered at the latest time point. These results suggest that while for *SLC38A2*, the major component of the repression is mRNA destabilization, ROMK1 was more translationally repressed, and perhaps the rebound in transcript levels represents an attempt by the cell to compensate for this reduction in protein levels. Others have reported activation of unknown feedback mechanisms for the recovery of gene expression^54^,^55^, which may account for the different directions of protein and mRNA levels.

Our DAVID analysis also showed where the miRNAs identified might exert regulatory functions; future studies will be required to perturb the steady state to address this further. Intriguingly, some of the signalling pathways enriched have been associated with chronic kidney disease (CKD) and the progression of glomerulosclerosis and tubulointerstitial and vascular fibrosis, particularly via transforming growth factor β-independent profibrotic pathways^56^. Also, ‘zinc finger hormone nuclear receptor’ was a GO term enriched in the analysis; specific nuclear binding sites for aldosterone, glucocorticoids and vitamin D are localized in the distal part of the nephron and can modulate carbohydrate and lipid metabolism, and regulate immune and inflammatory responses^57^. They are also known to be involved in the progression of fibrosis and are already being exploited as pharmacological targets in several metabolic diseases^58^. Finally, exosomes have recently been implicated in the development of metastasis^59^, and transcriptional regulation in cancer too was represented in the KEGG analysis.

Hence, on the basis of our data, we suggest that the presence of significant numbers of miRNAs in urinary exosomes might be indicative of potential regulatory roles these small RNAs might play in the kidney, as supported by both bioinformatic and renal epithelial cell studies. Further studies will be required to assess the impact of exosomal miRNAs on pathways that contain potential targets, as well to investigate urinary exosome miRNA profiling as a means for the identification of specific biomarkers of disease states.

## Methods

### Exosomal Isolation and confirmation

Under Cambridgeshire Research Ethics Committee approval (08-H0306-62) and with the informed consent of participants, exosomes were isolated from the second void urine of healthy volunteers as previously described^19^. All methods were performed in accordance with the relevant laboratory guidelines and institutional regulations. Briefly, samples were collected in sterilized bottles containing Complete EDTA-free protease inhibitor cocktail tablets, according to the manufacturer’s instructions. Immediately after collection, urine samples were centrifuged for 20 min at 17,000 × *g* at 4 ^o^C using a JA-17 fixed angle rotor in a Beckman AVANTI centrifuge. Supernatant was passed through a 0.22 μm filter membrane and ultracentrifuged for 120 minutes at 235,000 × *g* at 4 ^o^C in a Ti45 fixed-angle rotor, using a Beckman Optim ultracentrifuge. Exosomal pellets from individual samples were suspended in 100 μL of PBS while the final volume of pooled samples did not exceed 350 μL.

Pooled samples used for cell exposure and live cell imaging studies were washed 3 times, re-suspended in PBS and used immediately or stored at 4 ^o^C overnight, preserving the integrity of the isolated nanovesicles. Protein contents of exosomal samples and exosome-depleted urine were quantified, and the presence of CD63 and TSG101 as exosomal markers was confirmed by western blotting. To size the isolated nanovesicles, negative staining transmission EM was performed according to standard methods.

### Exosomal RNA Isolation

RNA extraction for individual and pooled samples of urinary exosomes was performed using miRNeasy Mini Kit with RNeasy MinElute Cleanup Kit according to manufacturer’s instructions. RNAs were eluted in 14 μl of RNAse-free water and miRNA concentrations were measured using the Agilent Bioanalyzer 2100 with a small RNA Chip.

### Small RNA library preparation and sequencing

The Illumina TruSeq Small RNA-seq Sample Preparation Kit was used according to manufacturer’s instructions. The template was total kidney RNA (as positive control) or the small RNA-enriched fraction purified from exosomes isolated as above from 6 different volunteers (3 male, 3 female); libraries were prepared with a unique index before pooling. Amplified libraries were resolved on a 6% Novex TBE PAGE gel. DNA fragments of 145 bp to 160 bp were eluted and concentrated by ethanol precipitation. Libraries were qPCR-quantified using a KAPA library quantification kit. Samples were diluted to 8 pM and sequenced on a MiSeq as 36 bp single end reads.

### Sequencing data analysis

Following adapter removal, the sequencing datasets were pre-processed using the FASTX-Toolkit (http://hannonlab.cshl.edu/fastx_toolkit/). Reads with at least 80% of bases at quality score >30 were kept for further processing, and no length threshold was set for the remaining reads. Stringent bowtie parameters were set to increase the accuracy and specificity of mapping including zero mismatches, and only a single genomic location was allowed per read. Reads mapped to the *E. coli* genome (MG1655 for *E. coli* K-12) were discarded. Alignments were done by Bowtie v1.0.1^60^. MicroRNAs and miRNA precursor annotations were downloaded from miRBase v19. The remaining reads were mapped to the human genome GRCh37/hg19. The annotation of hg19 was downloaded from the ENSEMBL database.

### miRNA target prediction and gene enrichment analysis

Predicted miRNA targets were downloaded from 5 tools/databases: miRBase, miRanda, PicTar, PITA and miRDB^28^,^29^,^30^,^31^,^32^. Co-expression scores were calculated as indicated in Gennarino *et al*.^61^ to indicate ranked likelihood of being a true target. Gene targets were analysed through enrichment analysis using the DAVID^33^ web service and the RDAVIDWebService^62^ BioConductor package in R. Targets were submitted in the form of their main protein product (UniProt Identifiers). Statistical evidence of enrichment was assessed by a modified Fisher’s Exact Test within DAVID with false discovery rate of 0.05 and Benjamini-Hochberg correction for multiple testing.

### PCR Amplification of cDNAs

cDNA was prepared by standard methods and PCR-amplified using gene-specific primers for *SLC38A2* and *KCNJ1.* Primer sequences are listed in Supplementary Table S6. To compare abundance of exemplar miRNAs, qRT-PCR analysis was performed using individual TaqMan miRNA Assays (Supplementary Table S7) according to manufacturer’s instructions. Expression levels were calculated as logarithmic values of 2^−ΔCt^ normalized to the small-nucleolar RNA (snoRNA) RNU6B. To evaluate gene expression of selected miRNA targets, total RNA was extracted from cultured HKC-8 or HCD cells using the miRNeasy mini kit, reverse transcribed using the SuperScript^®^ VILO cDNA Synthesis Kit and subjected to qRT-PCR using the SensiFAST™ SYBR^®^ Hi-ROX Kit. Gene expression levels were calculated using the ΔΔCt method.

### Cell culture and exposure

HKC-8 cells were cultured in Dulbecco’s Modified Eagle’s Medium (DMEM)/Nutrient F-12 Ham with 100 Units/ml of penicillin and 100 μg/ml of Streptomycin at 37 °C and 5% CO_2_. HCD cells were cultured as previously described^63^. 10^6^ cells were plated in 12-well cell culture plates and fed with FBS-free media to avoid other exosome sources. 10 μg of exosomal protein from a pool of at least 3 urine samples were added to 90–100% confluent wells, and lysates of the exposed cells were collected 6, 24 or 48 h later. Exposures were repeated using 3 different exosomal pools. Supplementary Table S8 list primary antibodies used for immunoblotting of selected targets in cell lysates; species-appropriate IRDye^®^ secondary antibodies were used and bands were visualized in an Odyssey Infrared Imaging System. ISS, Version 2.1 was used for densitometry analysis.

### Live cell imaging of cellular internalization of urinary exosomes

Washed pooled urinary exosomes were labelled with PKH67 Green Fluorescent Cell Linker Mini Kit, SIGMA according to manufacturer’s instructions. Dyed exosomes were washed twice with 1% BSA and ultracentrifuged, suspended in serum-free media, and utilised for the experiment on the same day. A parallel sample with no exosomes was labelled under the same conditions, and constituted the negative control. 10^6^ HCD cells were seeded in glass bottom culture dishes and incubated overnight; 70–80% confluent plates were washed with PBS and a 2 μg/ml solution of Hoechst 33258 was used to identify cell nuclei. Unbound dye was washed with Hank’s buffer and 1 ml of FBS free media was added to the cell culture plate. PKH67-labelled exosomes or PKH67 only (negative control) were added to Hoechst labelled HCD cells and images were recorded every 5 seconds in a Zeiss LSM780 Confocal Microscope at 37 ^o^C for approximately 2 h.

### Statistical analysis

Data were analysed with GraphPad Prism V5.01 and presented as mean ± SE. Differences at 48 h were compared using unpaired Student’s *t*-test.

## Additional Information

**How to cite this article:** Gracia, T. *et al*. Urinary Exosomes Contain MicroRNAs Capable of Paracrine Modulation of Tubular Transporters in Kidney. *Sci. Rep.* **7**, 40601; doi: 10.1038/srep40601 (2017).

**Publisher's note:** Springer Nature remains neutral with regard to jurisdictional claims in published maps and institutional affiliations.

## Supplementary Material

Supplementary Information

Supplementary Dataset 1

Supplementary Dataset 2

Supplementary Dataset 3

Supplementary Video 1

Supplementary Video 2

## Figures and Tables

**Table 1:** Most abundant urinary exosomal mature miRNAs and pre-miRNAs.

Rank	miRNA	CPM	Rank	pre-miRNA	CPM
1	**hsa-miR-10b-5p**	558287	1	**hsa-mir-30a**	418851
2	**hsa-miR-10a-5p**	191562	2	**hsa-mir-10b**	171532
3	**hsa-miR-30a-5p**	50322	3	**hsa-mir-30d**	132932
4	hsa-miR-26a-5p	28774	4	**hsa-mir-10a**	95206
5	hsa-miR-22-3p	21503	5	**hsa-mir-192**	30327
6	**hsa-miR-204-5p**	18471	6	**hsa-mir-204**	26862
7	hsa-miR-181a-5p	17499	7	hsa-mir-30e	23683
8	hsa-miR-27b-3p	14522	8	hsa-mir-151a	14407
9	**hsa-miR-30d-5p**	13272	9	hsa-mir-222	7655
10	**hsa-miR-192-5p**	7684	10	hsa-mir-200b	7278

CPM, counts per million; species in **bold** are common to both lists.

**Table 2:** DAVID enrichment analysis of predicted targets of the 10 most abundant miRNAs.

Term	P value	Fold Enrichment	Adjusted P value*
Serine/threonine protein kinase	7.8E-10	2.1	1.3E-06
Zinc finger, nuclear hormone receptor	2.5E-06	3.9	4.2E-03
ErbB signalling pathway	8.8E-06	2.8	1.1E-02
PDGF signalling pathway	1.3E-05	2.0	1.4E-02
EGFR signalling pathway	2.2E-05	3.7	2.2E-02
Rho GTPase signalling pathway	2.9E-05	2.5	2.9E-02
T cell receptor signalling pathway	3.6E-05	2.5	4.4E-02

*Benjamini-Hochberg corrected False Discovery Rate.

**Table 3:** KEGG Pathway analysis for predicted targets of the 10 most abundant miRNAs.

Rank	miRNA	KEGG Pathway (all p ≤ 0.04)
1	hsa-miR-10b-5p	Transcriptional misregulation in cancer
2	hsa-miR-10a-5p	Transcriptional misregulation in cancer
3	hsa-miR-30a-5p	cGMP/PKG signallingAldosterone-regulated sodium reabsorptionUbiquitin mediated proteolysis
4	Has-miR-26a-5p	Proximal tubule bicarbonate reclamationFocal adhesion
5	hsa-miR-22-3p	EndocytosisBacterial invasion of epithelial cellsFc gamma R-mediated phagocytosis
6	hsa-miR-204-5p	LysosomeEndocrine and factor-regulated calcium reabsorption
7	hsa-miR-181a-5p	Estrogen signallingRenal cell carcinomaProstate cancer
8	hsa-miR-27b-3p	GnRH signallingTNF signalling
9	hsa-miR-30d-5p	Mucin type O-Glycan biosynthesisUbiquitin mediated proteolysis
10	hsa-miR-192-5p	RNA degradation,Type I diabetes mellitus
