# Supplementary material for: Urinary Exosomes Contain MicroRNAs Capable of Paracrine Modulation of Tubular Transporters in Kidney
Source: Sci Rep. 2017 Jan 17;7:40601. doi: 10.1038/srep40601 (PMC5240140; doi:10.1038/srep40601)

Urinary Exosomes Contain MicroRNAs Capable of Paracrine Modulation of Tubular Transporters in Kidney

SUPPLEMENTARY DATA

Tannia Gracia, Xiaonan Wang, Ya Su, Elizabeth E. Norgett, Timothy L. Williams, Pablo Moreno, Gos Micklem and Fiona E. Karet Frankl

Supplementary Figure 1. Characterization of exosomes isolated from urine

A

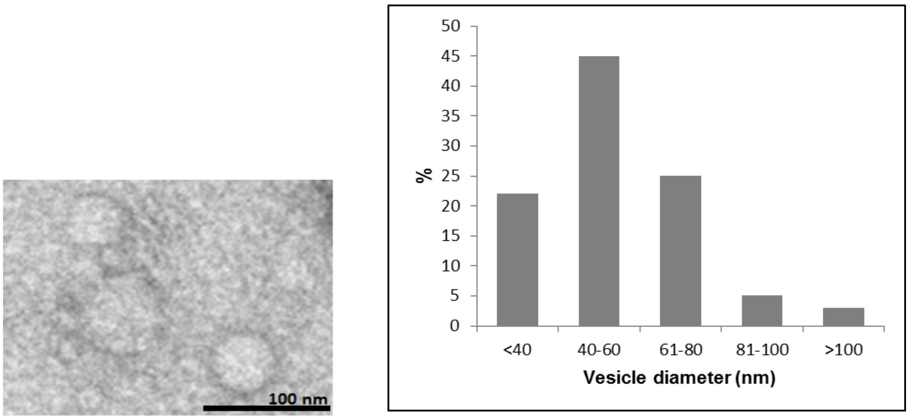

B

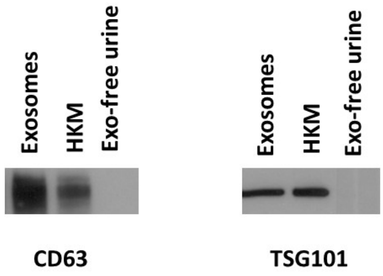

(A) Electron microscopy shows the integrity of urinary exosomes: rounded vesicles with a size distribution of 59.0±1.93 nm. **(B)** The presence of exosomal markers CD63 and TSG101 was confirmed by western blot. Both markers were absent in exosome-free urine. HKM = Human Kidney Membrane.

Supplementary Figure 2. Confirmation of expression of selected predicted miRNA targets in human proximal tubular and human collecting duct cells

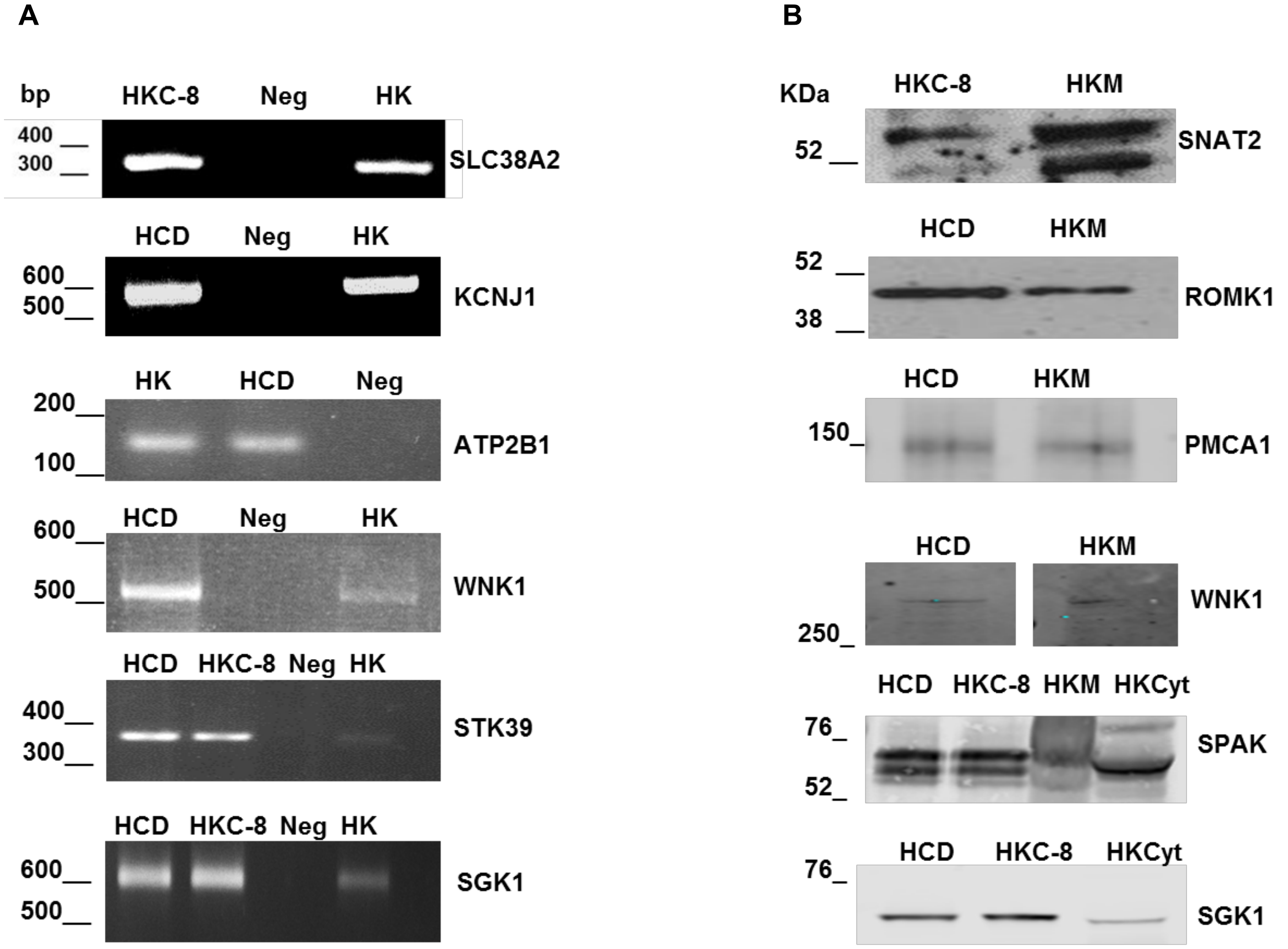

Panels in column A represent RT-PCR products corresponding to mRNA expression of miRNA predicted targets in HKC-8 (human proximal tubular) and HCD (human collecting duct) cells. Negative control template was water and positive control template was human kidney cDNA. Panels in column B are western blots of HKC-8 and HCD cell lysates using antibodies against the selected targets. Positive controls were human kidney membrane (HKM) or cytosol (HKcyt). bp= base pair, KDa=kilodalton

Supplementary Figure 3. Buffer alone or UMOD do not affect expression levels in HCD and HKC8 cells respectively

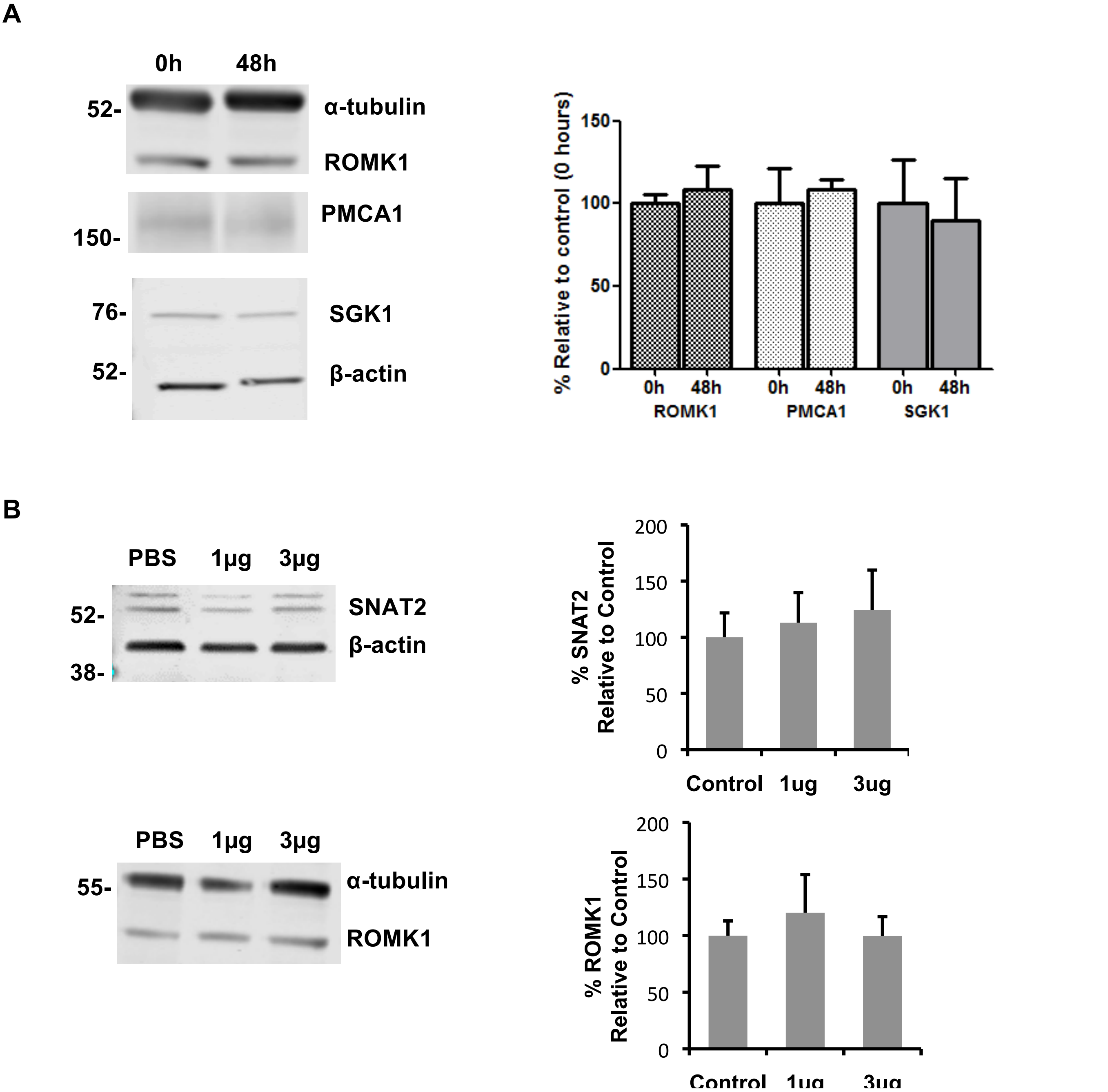

(**A**) Buffer alone does not alter protein levels in this cell culture system: representative western blots and densitometry of ROMK1, PMCA1 and SGK1, in HCD cells exposed to buffer (PBS) for 48 hours are shown. PMCA1 was detected on the same blot as ROMK1, with α-tubulin as the loading control. (**B**) Representative western blots and densitometry of SNAT2 in HKC-8 cells and ROMK1 in HCD cells exposed to PBS (Control), 1µg or 3 µg of uromodulin for 48h. No significant differences were observed. All densitometry analyses are of 3 repeats.

Supplementary Table 1. miRNA amount extracted from urinary exosomes of individual filtered urine samples

Sample No.	Volume (mL)	miRNA ng/100mL Urine	miRNA (% of small RNAs)
HV003F	380	4.6	65
HV049F	250	15.8	52
HV055F	450	3.1	44
HV060F	220	9.1	49
HV061F	250	13.5	87
HV056M	300	6.4	41
HV057M	380	5.6	63
HV059M	380	13.0	49
HV064M	300	1.3	30
HV075M	300	1.0	24
**Mean**	**321**	**7.3**	**50.40**
**SE**	**23.26**	**1.67**	**5.73**

F = Female, M = Male

Supplementary Tables 2-4: provided separately

Supplementary Table 5. miRNA amount extracted from exosomes isolated from filtered and non-filtered samples

Sample	Volume	Filtered	Not Filtered
No.	mL	miRNA(ng/100mL Urine)	
HV003F	200	3.8	16.0
HV049F	125	3.0	10.0
HV065F	225	0.5	3.8
HV056M	200	3.3	42.4
HV064M	150	1.3	5.5
HV057M	225	1.1	2.7

F = Female, M = Male

Supplementary Table 6. Primer sequences used for PCR and qRT-PCR Amplification of cDNAs of selected targets

Gene	Forward primer sequence [5'3']	Reverse primer sequence [5'3']	Amplicon Length [bp]
KCNJ1	ATTGTGATCCCACAAGACATGC	CAACTCCTCATTGCTGTCTTCG	587
STK39	CAGTGAGTGCCAGCACCATC	CAGCTGACACTCAACTGAGC	391
SGK1	TATGACAGGACTGTGGACTG	AAGGCGGCACTCTAACGCTC	635
WNK1	ATGCCATGAATCTCAGGCAG	AGACTCTCCATTCTGAGGGCTC	542
ATP2B1	CCTGAGGAGGAATTAGCAGAGGA	CTACGAAATGCATTCACCACTCGAAT	125
SLC38A2	GTCATAGTCTCATTACAGTGTC	CTGGCATCAGATGGACTGAG	328
	**qRT-PCR**		
SLC38A2	GTCATAGTCTCATTACAGTGTC	CACCAACTTGATATAGAAGGC	NA
KCNJ1	CACCAACTTGATATAGAAGGC	CTTCATCCTGGCTCTAACAT	NA
B2M	GAGGCTATCCAGCGTACTCCA	CGGCAGGCATACTCATCTTTT	NA
GAPDH	GGAGCGAGATCCCTCCAAAAT	GGCTGTTGTCATACTTCTCATGG	NA

Supplementary Table 7. TaqMan® MicroRNA Assays used to compare miRNA abundance by qRT-PCR

Assay ID	miRNA	Sequence
000388	hsa-miR-10b	UACCCUGUAGAACCGAAUUUGU
000387	hsa-miR-10a	UACCCUGUAGAUCCGAAUUUGUG
000417	hsa-miR-30a-5p	UGUAAACAUCCUCGACUGGAAG
000470	hsa-miR-148a	UCAGUGCACUACAGAACUUUGU
000474	hsa-miR-152	UCAGUGCAUGACAGAACUUGGG
001093	RNU6B	CGCAAGGAUGACACGCAAAUUCGUGAAGCGUUCCAUAUUUUU

Supplementary Table 8. Antibodies used for immunoblotting

Antibody Target/Name	Source/Catalogue number	Type	Dilution
	**Primary Antibodies**		
CD63	Abcam/ab8219	Mouse/mAb	1:100
TSG101	Abcam/ab83	Mouse/mAb	1:100
ROMK1	SIGMA/SAB2501215	Goat/pAb	1:100
SPAK	Abcam/ab128894	Rabbit/mAb	1:200
SGK1	Abcam/ab43606	Rabbit/pAb	1:100
WNK1	Abcam/ab53151	Rabbit/pAb	1:100
PMCA1	Abcam/ab190355	Rabbit/mAb	1:100
SNAT2	SIGMA/AV33058	Rabbit/pAb	1:100
Uromodulin	CEDERLANE/CL1032A	Mouse/mAb	1:200
β-Actin	Abcam/ab6276	Mouse/mAb	1:2000
α-Tubulin	Abcam/ab7291	Mouse/mAb	1:2000
	**Secondary Antibodies**		
IRDye® 680 RD	925-68073	Donkey anti-Rabbit	1:1000
IRDye® 800 CW	926-32214	Donkey anti-Goat	1:1000
IRDye® 800 CW	926-32210	Goat anti-Mouse	1:2000

mAb = monoclonal antibody; pAB = polyclonal antibody

Additional material

Video 1

Live cell microscopy of Hoescht-333-labelled human collecting duct (HCD) cells exposed to PKH67-labelled exosomes (green) over a 2h period, showing adherence and progressive internalisation.

Video 2

Control live cell microscopy of similar HCD cells exposed to PKH67 alone over a 2h period.

Additional Material for Figures 4/5

ORIGINAL BLOTS FOR FIGURE 4

Blot for Figure 4A

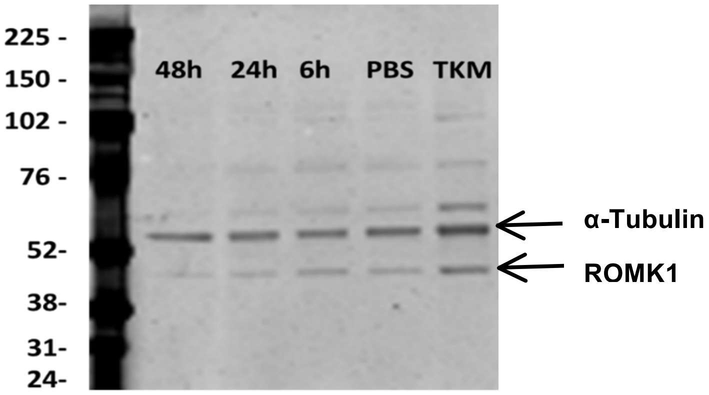

Blot for Figure 4B

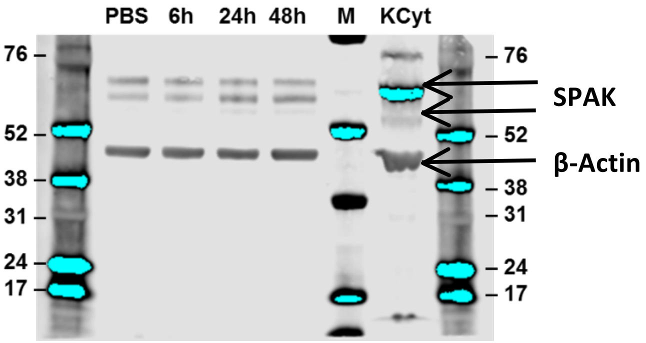

β-Actin : 45KDa
α-Tubulin : 55 KDa
ROMK1: 45KDa
SPAK: 60KDa, 65KDa
SGK1: 59 KDa
WNK1: 250KDa, 225KDa
TKM: Total Kidney Membrane
KCyt: Kidney Cytosol
M: Marker (Rainbow, Amersham)

Blot for Figure 4C

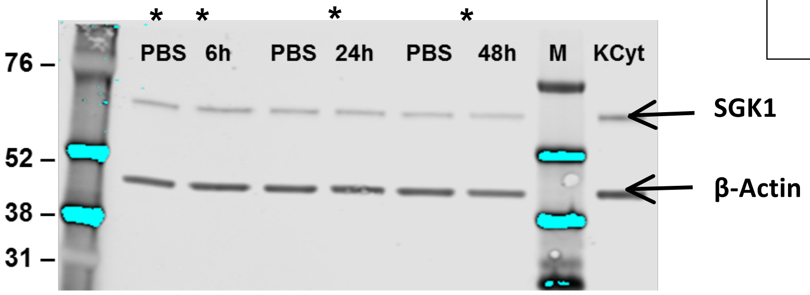

Blot for Figure 4D

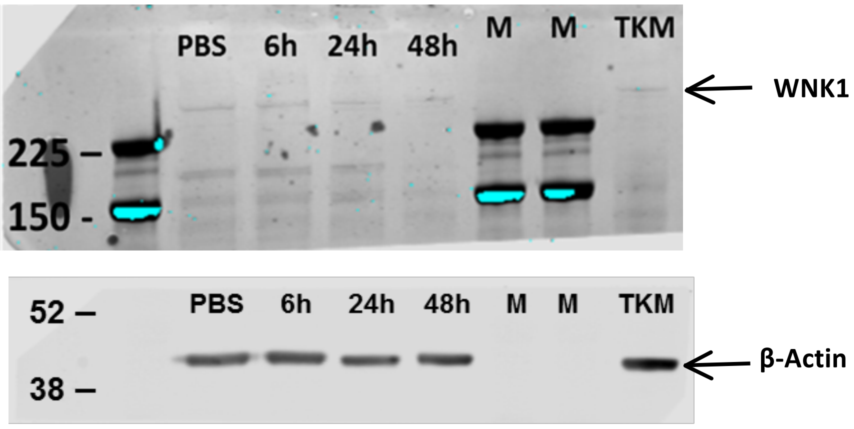

Blot for Figure 4E

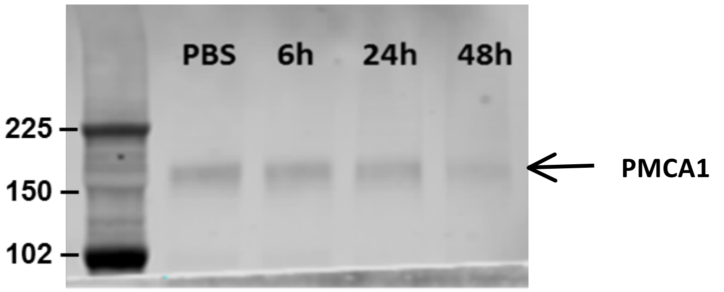

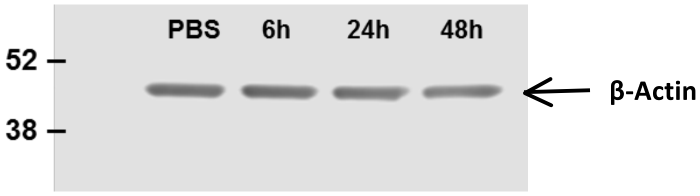

ORIGINAL BLOTS FOR FIGURE 5

PMCA1: 140-150 KDa
β-Actin : 45KDa
α-Tubulin : 55 KDa
SNAT2: 55KDa
SGK1: 59 KDa
M: Marker (Rainbow, Amersham)
***** **:** Portion of the blot used for Figure 5A

Blot for Figure 5A

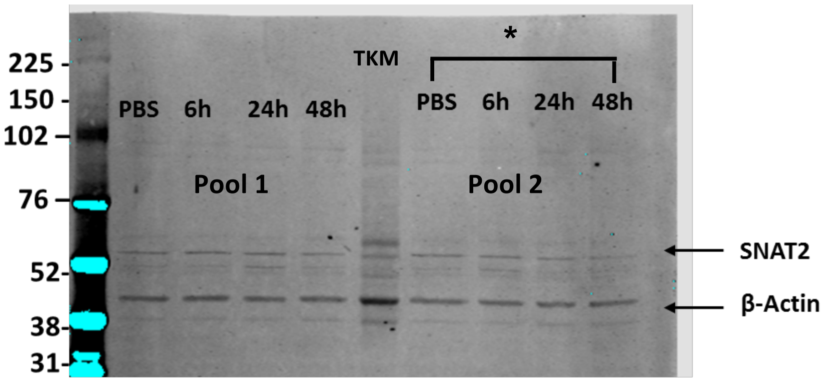

Blot for Figure 5B

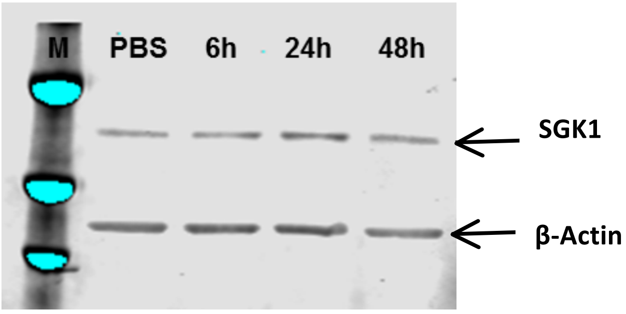

Supplement: Supplementary Information [file srep40601-s1.pdf]
